# Retrospective Review of Children Hospitalized for Epstein–Barr Virus-Related Infectious Mononucleosis

**DOI:** 10.3390/pathogens14070702

**Published:** 2025-07-16

**Authors:** Shufeng Tian, Jinjun Zheng, Zhe Zhou, Qingluan Yang, Biao Sun, Yuxi Li, Zengrui Lin, Yuchun Long, Song Guan, Sen Wang, Jiexin Zhuang, Wenhong Zhang, Lingyun Shao, Jikui Deng

**Affiliations:** 1 Department of Infectious Diseases, Shenzhen Children’s Hospital, Shenzhen 518038, China; szsetyytsf@163.com (S.T.); zjj13690145347@163.com (J.Z.); xiaosun0225@163.com (B.S.); szetyylyx@163.com (Y.L.); linzengrui@hotmail.com (Z.L.); long977459@126.com (Y.L.); 2310244240@email.szu.edu.cn (S.G.); 2 Department of Infectious Diseases, Shanghai Key Laboratory of Infectious Diseases and Biosafety Emergency Response, National Medical Centre for Infectious Diseases, Huashan Hospital, Shanghai Medical College, Fudan University, No. 12, Urumqi Middle Road, Jing’an District, Shanghai 200040, China; 20211220006@fudan.edu.cn (Z.Z.); qlyang10@fudan.edu.cn (Q.Y.); sen.wang@nmcid.org.cn (S.W.); wenhongzhang_hs@126.com (W.Z.); 3 Shanghai Sci-Tech Inno Centre for Infection and Immunity, Room 201, No. 6, Lane 1220, Huashan Road, Changning District, Shanghai 200052, China; 4 Department of Pediatrics, Longhua Branch of Shenzhen People’s Hospital, Shenzhen 518109, China; zjx06408@gmail.com; 5 Institute of Infection and Health, Fudan University, Shanghai 200032, China

**Keywords:** Epstein-Barr virus, infectious mononucleosis, children, prolonged fever, abnormal liver function, treatment outcomes

## Abstract

Objectives: Our objective was to investigate the clinical characteristics, complications, and treatment outcomes of Epstein–Barr virus (EBV)-related infectious mononucleosis (IM) in children and to identify risk factors associated with prolonged fever and abnormal liver function. Methods: This retrospective study included 3006 children admitted to Shenzhen Children’s Hospital from May 2009 to April 2024 with suspected EBV-related IM. After excluding cases without etiological evidence and those with underlying diseases, 2660 cases were analyzed. Data on demographics, clinical manifestations, laboratory findings, complications, and treatment outcomes were collected. Logistic regression was used to identify risk factors for prolonged fever and abnormal liver function. Results: Among the 2660 confirmed cases, patients ranged from 8 months to 17 years of age, with a median age of 4 years and a male-to-female ratio of 1.46:1. Co-infections were identified in 369 (13.9%) patients, predominantly with Group A Streptococcus. Complications occurred in 560 (24.46%) of the 2289 patients without co-infections, with bronchitis being the most common (42.68%). Elevated ferritin and atypical lymphocyte percentage were associated with prolonged fever (*p* < 0.001), while elevated lactate dehydrogenase (LDH) and a lower CD4% predicted abnormal liver function (*p* < 0.001). Antiviral therapy did not shorten fever duration or hospital stay but prolonged both when combined with corticosteroids or intravenous immunoglobulin (IVIG) (*p* < 0.001). Conclusions: Specific laboratory markers such as ferritin, atypical lymphocyte percentage, LDH, and CD4% are important predictors of prolonged fever or liver dysfunction in EBV-IM. Our findings suggest that antiviral therapy may not be beneficial in uncomplicated cases and highlight the need for tailored treatment strategies to optimize patient outcomes.

## 1. Background

The Epstein–Barr virus (EBV), also known as Human Herpesvirus 4, is a ubiquitous double-stranded DNA virus found in approximately 95% of the global population [1,2]. Infectious mononucleosis (IM) is a common clinical manifestation of primary EBV infection that was first described by Sprunt and Evans in 1920 [3]. Primary EBV infection can occur at any age but often presents as IM in both preadolescents and adolescents or young adults, often resulting in IM, which is characterized by fever, pharyngitis, lymphadenopathy, hepatosplenomegaly, and atypical lymphocytosis [4,5]. Although most cases of IM resolve spontaneously with the development of EBV-specific immunity [6], there is significant variation in disease progression and complications across different populations.

EBV-IM is generally self-limiting, but its potential for severe complications warrants heightened vigilance [7,8]. In a nationwide retrospective study conducted in China, it was observed that EBV-IM is prevalent, with a significant number of cases requiring hospitalization due to severe symptoms [9]. Furthermore, co-infections with other pathogens can exacerbate the severity of EBV-related disease. For instance, cytomegalovirus (CMV) co-infection can impair immune function and compromise the immune control of EBV, thereby worsening clinical outcomes [10]. Recent studies have underscored the significance of mixed infections involving EBV and other pathogens in pediatric populations [11]. These studies indicate that EBV co-infection with other viruses, bacteria, or parasites can significantly impact disease severity. Notably, EBV co-infection with Helicobacter pylori (HP) has been associated with more severe inflammatory responses and an increased risk of developing gastric cancer [12]. Collectively, these findings highlight the complex interactions between EBV and other pathogens, which can have profound clinical implications.

Given the multifaceted nature of EBV infection and its impact on pediatric health, our study aims to provide a comprehensive exploration of the clinical characteristics, mixed infections, comorbidities, and laboratory findings associated with IM. Specifically, we will analyze the prevalence and clinical significance of mixed infections involving EBV and other pathogens, as well as the distribution and impact of comorbidities on disease severity and prognosis. We seek to identify factors contributing to prolonged fever and liver function impairment by examining the associations between clinical manifestations and inflammatory markers. Additionally, we aim to evaluate the efficacy and safety of current treatment strategies to offer evidence-based recommendations for the management of IM. Through this research, we hope to bridge the gap between clinical observations and scientific understanding, ultimately improving outcomes for patients affected by this common yet complex viral illness.

## 2. Methods

### 2.1. Study Participants

The study was a retrospective observational analysis at Shenzhen Children’s Hospital, the sole tertiary-level children’s hospital in Shenzhen, Guangdong Province, China, with a capacity of 1300 beds [13]. Serving a population of 17.56 million, the hospital is the largest pediatric specialty facility in the province and offers advanced medical services, education, and research. Its unique position as Shenzhen’s only children’s hospital lends the study a representative significance. We collected data from 3006 children with EBV-IM who were hospitalized for treatment at the Shenzhen Children’s Hospital from May 2009 to April 2024. “Hospitalized” was defined as an admission with a minimum stay of 24 h, based on clinical necessity and medical records.

### 2.2. Inclusion and Exclusion Criteria

**Figure 1:**
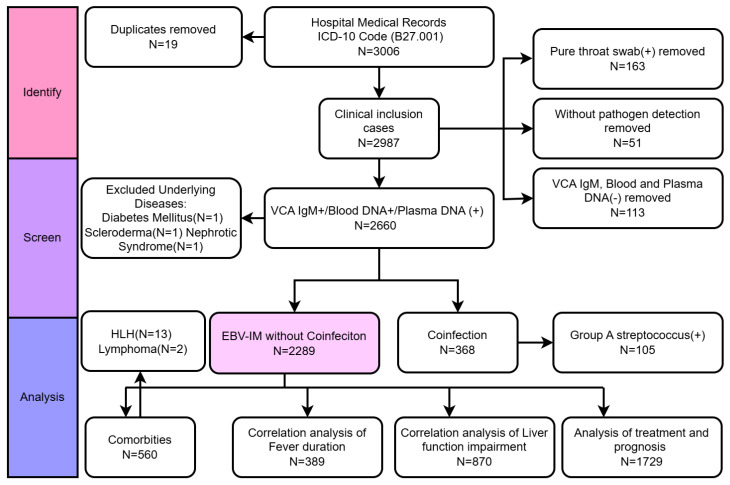
Flowchart of study participants. Abbreviations: HLH, hemophagocytic lymphohistiocytosis; EBV, Epstein–Barr virus; IM, infectious mononucleosis.

This retrospective study included only children under the age of 18 who were hospitalized for IM according to the tenth revision of the International Statistical Classification of Diseases and Related Health Problems 10th Revision (ICD-10) code B27.001 (Figure 1). Children were diagnosed with IM according to the clinical and etiological diagnosis based on the criteria outlined in “Zhu Futang Practical of Pediatrics” [14]. Complete clinical data are available, excluding cases of malignant tumors, underlying kidney diseases, or those without pathogen support (positive for VCA IgM and/or blood EBV DNA) [15,16].

### 2.3. Clinical Data Collection

All clinical data were collected from the hospital medical records. This study collected the following data: age, gender, clinical manifestations, complete blood count results, biochemical panel (including liver function indices), cellular immune function, and treatment situation.

For patients with IM caused solely by EBV infection without co-morbid infections or underlying diseases, we analyzed their co-morbidities, treatments, and prognosis. Additionally, we conducted an analysis of factors related to liver function damage and fever duration (Figure 1).

When the aspartate aminotransferase (AST) or alanine aminotransferase (ALT) is elevated by two or more times the upper limit of reference, without other causes of elevated AST or ALT, it can be diagnosed that the existence of IM is complicated by hepatic dysfunction [9]. Fever was defined as stated history or presence of fever of ≥37.5 °C. To identify risk factors contributing to abnormal liver function (LFT) and prolonged fever, as these manifestations are clinically significant. Prolonged fever was defined as a fever lasting more than one week. Abnormal LFT and prolonged fever have been associated with increased morbidity in patients with infectious diseases.

Cellular immune function was assessed by flow cytometry, including the percentage of CD4+ T lymphocytes (CD4%) in peripheral blood lymphocytes.

### 2.4. Pathogen Detection

In this retrospective analysis, the presence of pertinent clinical symptoms combined with positive results from nucleic acid testing, culture, or antigen testing can be used to identify a mixed infectious agent. Detailed diagnostic methodologies for all pathogens are provided in Supplementary Table S1.

### 2.5. Statistical Analysis

IBM SPSS Statistics 23.0 software (SPSS Inc., Chicago, IL, USA) was used for data analysis. Continuous variables were expressed as mean ± SD if normally distributed, or median [Q1, Q3] if non-normally distributed, and categorical variables as frequency (%). Chi-squared tests and Fisher’s exact tests were used for categorical variables, while one-way ANOVA was used for normally distributed continuous variables and the Kruskal–Wallis test for non-normally distributed continuous variables. Categorical variables were expressed as number (%) or proportions and compared between/among groups by χ^2^ or Fisher’s exact tests, when appropriate. Logistic regression analysis was used to assess the risk factors for liver function damage and fever duration exceeding one week in children with infectious mononucleosis. The receiver operating characteristic (ROC) curve was applied to determine the cutoff value of atypical lymphocyte ratio in patients with fever duration exceeding one week. A *p* < 0.05 (2-tailed) was considered statistically significant.

## 3. Results

### 3.1. Characteristics of the Study Population

Between May 2009 and April 2024, Shenzhen Children’s Hospital admitted 3006 inpatients with EBV-IM. After removing duplicates and cases lacking etiological evidence, 2660 children were confirmed with EBV-IM, including 3 with underlying diseases (Figure 1). Among these confirmed cases, the distributions of Epstein–Barr virus (EBV) capsid antigen immunoglobulin M (VCA IgM) and EBV DNA polymerase chain reaction (PCR) test results were as follows: 1842 patients (69.25%) were positive for both VCA IgM and PCR, indicating a typical acute EBV infection; 557 patients (20.94%) were PCR-positive but VCA IgM-negative; and 261 patients (9.81%) were PCR-negative but VCA IgM-positive.

In total, 2660 inpatients ranged from 8 months to 17 years old, with a median age of 4 years (Table 1); most were aged 1–6 years (Table 1). The gender ratio was 1578 males to 1082 females (1.46:1). The median hospital stay was 5 days, mostly between 3 and 7 days (59.7%), and the median fever duration was 7 days, with 52.4% experiencing fever for less than 7 days (Table 1). Among 2285 patients (Figure 1) without co-infections or underlying diseases, 859 (37.53%) developed liver function impairment.

### 3.2. Main Clinical Manifestations of Inpatients

**Figure 2:**
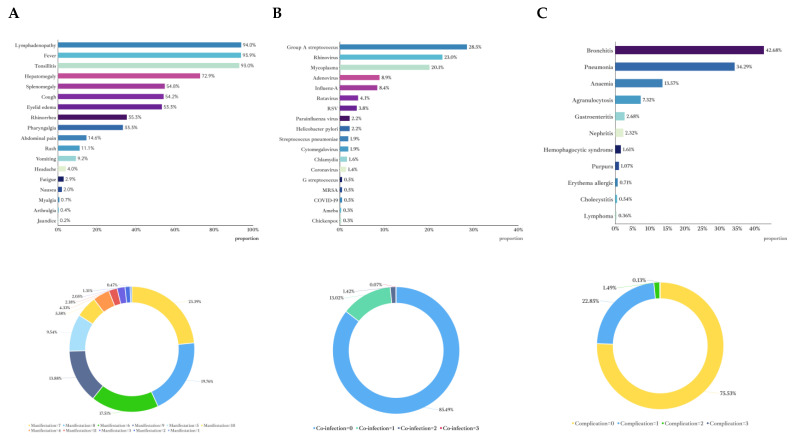
Clinical features of the inpatients with EBV-associated infectious mononucleosis include the clinical manifestations (**A**), distribution of mixed infections (**B**), and complications (**C**).

The characteristics of clinical manifestations, complications, co-infections with other pathogens, and duration of hospitalized EBV- IM were clearly identified (Figure 2A–C and Supplementary Table S2). The most common clinical features included fever (93.9%), tonsillitis (93%), lymphadenopathy (94%), and hepatomegaly (72.9%). Other frequent symptoms were cough (54.2%), rhinorrhea (35.3%), and pharyngalgia (33.3%). Less common symptoms included fatigue (2.9%), nausea (2.0%), and jaundice (0.2%).

Note: The detection methods for the pathogens listed are as follows: GAS is identified using throat swab antigen tests, Helicobacter pylori via carbon-13 urea breath tests, rotavirus through stool antigen tests, cytomegalovirus by blood nucleic acid tests, varicella zoster virus by nucleic acid tests of vesicle fluid, and amoebae by routine microscopic examination of fecal samples. MRSA and group G Streptococcus are identified through sputum culture. All other pathogens were detected using nasopharyngeal swab nucleic acid tests; more detail in Supplementary Table S1.

### 3.3. Co-Infection

This study included 2660 patients, among whom 369 (13.9%) had co-infections (including one patient with an underlying condition; Figure 2B). Of these co-infected patients, 40 (10.8%) had multiple pathogens. The distribution of co-infecting pathogens was as follows: group A streptococcus (105 cases, 28.5%) was the most common pathogen, followed by rhinovirus (85 cases, 23.0%) and mycoplasma (74 cases, 20.3%). Other frequently detected pathogens included adenovirus (33 cases, 8.9%), influenza A virus (31 cases, 8.4%), rotavirus (15 cases, 4.1%), and others. Additionally, less common pathogens such as coronavirus, group G streptococcus, methicillin-resistant staphylococcus aureus, SARS-CoV-2, entamoeba histolytica, and varicella zoster virus were also identified. Overall, co-infections were predominantly caused by bacterial and common respiratory viral pathogens, with a notable proportion of multiple infections, necessitating heightened clinical attention.

The results indicate no significant difference in liver function abnormality rate between uncomplicated EBV-IM (50.32%) and co-infection (46.7%) groups (*p* = 0.212) nor within the GAS subgroup (56.2%, *p* = 0.838). Fever duration was significantly longer in the co-infection group (7 days, IQR 5–10) compared to the uncomplicated EBV-IM group (7 days, IQR 5–9) (*p* < 0.001), with the GAS subgroup showing a shorter duration (6 days, IQR 5–10) (*p* < 0.001). Hospital stay was significantly longer in the co-infection group (5 days, IQR 4–7) (*p* = 0.017) than in the uncomplicated EBV-IM group (5 days, IQR 4–6), with no significant difference in the GAS subgroup (5 days, IQR 4–7) (*p* = 0.217). More details can be found in Supplementary Table S3.

### 3.4. Prevalence of Complications

After excluding 368 patients with mixed infections and an additional 3 patients with underlying diseases, 2289 patients were included in the analysis of complications. Among these, 560 patients (24.46%) were identified as having at least one complication. The most prevalent complication was bronchitis, affecting 42.68% of the patients with complications. Pneumonia was the second most common complication, which was observed in 34.29% of the patients. Anemia was also a significant issue, being present in 13.57% of the cases. Other notable complications included agranulocytosis (7.32%), gastroenteritis (2.68%). and nephritis (2.32%). Less frequent complications included hemophagocytic syndrome (1.61%), purpura (1.07%), erythema allergic (0.71%), cholecystitis (0.54%), and lymphoma (0.36%). The pie chart depicts the distribution of co-morbidities among the patients, indicating that patients with multiple complications represent 1.51% of the total cases (Figure 2C).

### 3.5. Risk Factors for Prolonged Fever and Abnormal Liver Function

Multivariate logistic regression analysis identified several risk factors associated with fever duration ≥7 days and abnormal liver function. Elevated ferritin levels (Table 2, *p* < 0.001) and higher percentages of atypical lymphocytes (Table 2, *p* = 0.012) were significantly associated with prolonged fever. For abnormal liver function, elevated lactate dehydrogenase (LDH) levels (OR: 1.004, 95% CI: 1.002–1.005, *p* ≤ 0.001), C-reactive protein (CRP) levels (OR: 0.955, 95%CI: 0.935, 0.976, *p* ≤ 0.001), and a lower CD4% (OR: 0.894, 95% CI: 0.856–0.935, *p* ≤ 0.001) were significant predictors.

**Figure 3:**
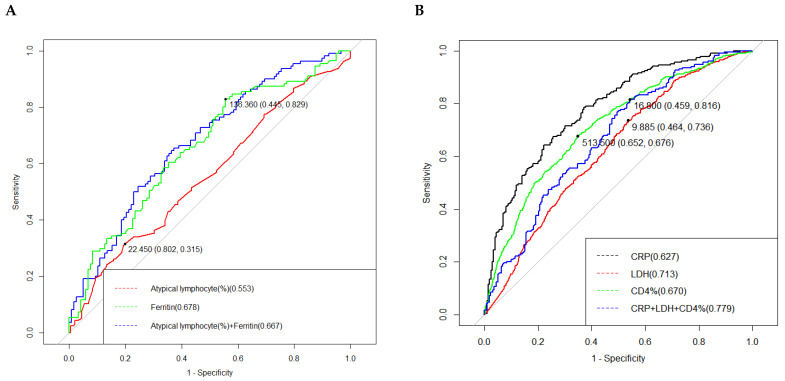
ROC curves for predicting fever duration over one week (**A**) and liver function impairment (**B**) in infectious mononucleosis. Different colors indicate the type of biomarker as shown in the inset legend. AUC values, sensitivity, specificity, PPV, and NPV for biomarkers are described in the Supplementary Table S4. Abbreviations: ROC, receiver operating characteristic; AUC, area under the curve; PPV, positive predictive value; NPV, negative predictive value; CRP, C-reactive protein; LDH, Lactate dehydrogenase; CD4%, CD4-positive T-lymphocyte percentage.

The figure below shows the receiver operator characteristic (ROC) curves for predicting fever duration over one week (Figure 3A). The area under the curve (AUC) was 0.679 (95% CI: 0.607–0.746) for the combined model of atypical lymphocyte percentage and ferritin levels. The sensitivity and specificity of the combined model were 62.5% and 62.7%, respectively.

The ROC curve for predicting liver function impairment (Figure 3B). The AUC was 0.779 (95% CI: 0.735–0.823) for the combined model of CRP, LDH, and CD4%. The sensitivity and specificity of the combined model were 58.7% and 85.2%, respectively.

### 3.6. WBC and EBV DNA Load Analysis in Patients with HLH, Prolonged Fever, and Liver Function Impairment

Preparation of EBV DNA loads in whole blood and plasma (Supplementary Table S6), alongside white blood cell (WBC) counts, was conducted across patient subgroups defined by complications and clinical features (Table 3). Patients with HLH had significantly lower WBC counts (median 4.06 × 10^9^/L) compared to IM patients (median 14.46 × 10^9^/L, *p* = 0.015). There was no significant difference in the EBV DNA loads between HLH and IM groups. Fever duration did not significantly affect the EBV DNA levels or WBC counts. Abnormal liver function was associated with higher whole-blood EBV DNA loads (340.50 vs. 187.00 × 10^3^ copies/mL; *p* = 0.001) and slightly elevated WBC counts (14.00 vs. 13.40 × 10^9^/L; *p* = 0.002), while plasma EBV DNA levels showed no significant difference (*p* = 0.085) despite a higher median in the abnormal group (3.73 vs. 2.88 × 10^3^ copies/mL).

### 3.7. Treatments

After excluding patients with co-infections and comorbidities, a total of 1729 patients with uncomplicated IM were included in this study. The treatment approaches are summarized in Supplementary Table S5. The majority of patients (45.81%) received symptomatic support treatment without any medication. Acyclovir was used in 49.33% of patients, with 42.44% receiving acyclovir monotherapy. The use of corticosteroids and intravenous immunoglobulin (IVIG) was less common, at 7.23% and 8.21%, respectively. Four treatment regimens were compared: acyclovir monotherapy, symptomatic support treatment without any medication, acyclovir combined with corticosteroids, and acyclovir combined with IVIG. The results showed that the average duration of fever was 7 (5–9) days in patients without any medication, which was not significantly different from the acyclovir monotherapy group (*p* > 0.05). However, the fever duration was significantly prolonged in patients receiving IVIG or corticosteroids (*p* < 0.001). Additionally, the hospitalization duration was significantly longer in patients receiving antiviral therapy (*p* < 0.001), with a median of 5.0 (4.0–7.0) days compared to 4 (3–6) days in those without medication. The combination of antiviral therapy with corticosteroids or IVIG further extended the hospitalization duration to 6 (5–7) days and 8 (6–10) days, respectively, which was significantly different from the group without medication (*p* < 0.001).

## 4. Discussion

This large-scale retrospective study delineates key predictors of severe outcomes in children hospitalized with EBV-associated IM. We identified elevated serum ferritin and atypical lymphocyte percentage as independent risk factors for prolonged fever (≥1 week), while elevated LDH and reduced CD4% were significant predictors of abnormal liver function. Furthermore, co-infections, particularly with group A streptococcus (GAS), were associated with extended fever duration and hospital stay. Crucially, antiviral therapy (acyclovir), especially when combined with corticosteroids or intravenous immunoglobulin (IVIG), not only failed to shorten fever duration or hospitalization but was associated with a significant prolongation of both.

This study identified elevated serum ferritin and increased atypical lymphocyte proportion as independent predictors of prolonged fever (≥1 week) in pediatric EBV-IM. Critically, ferritin >136.36 ng/mL (ROC-derived cutoff; Figure 3A) provided optimal discrimination for extended febrile courses, reinforcing its clinical utility. This threshold likely represents the inflection point where ferritin’s dual roles as an acute-phase reactant and macrophage activation marker transition into pathological significance—signifying systemic hyperinflammation or subclinical HLH predisposition that perpetuates fever through cytokine-mediated pathways [17]. The defined cutoff enables rapid risk stratification: patients exceeding this level at presentation warrant intensified monitoring for evolving hyperinflammatory complications. Concurrently, atypical lymphocytes—morphological indicators of EBV-specific cytotoxic T-cell expansion—reflect antiviral immune activity that may become dysregulated. While essential for viral control, excessive activation perpetuates tissue inflammation and prolongs symptoms [18,19]. Critically, an atypical lymphocyte proportion >22.45% (ROC-derived cutoff; Figure 3A) optimally identified patients at risk for fever duration ≥1 week (AUC = 0.679). This threshold provides immediate clinical utility: detection via routine blood smear enables rapid, cost-effective risk stratification and facilitates objective communication regarding anticipated illness course.

Liver function impairment is a complex pathological process influenced by multiple factors. In our study, among hospitalized children with EBV-IM, liver function impairment was a common acute manifestation, occurring in 37.53% of cases. Elevated levels of CRP and LDH have been shown to correlate with the severity of liver function impairment and can serve as useful auxiliary tools for assessing the condition [20]. Additionally, a decrease in the CD4% is considered an important indicator of liver function impairment. CD4+ T cells play a crucial role not only in mediating direct cytolytic responses but also in orchestrating polyfunctional immune reactions and modulating viral latency states. These functions are essential for long-term EBV containment and immune response modulation [21,22,23]. The development of EBV-specific immunity is vital for resolving IM symptoms. During the initial infection, the body mounts a robust immune response, characterized by the activation of both CD4+ and CD8+ T cells targeting EBV antigens [24]. This immune response is critical for controlling the viral load and facilitating recovery from the acute phase of the disease [25]. Furthermore, higher levels of EBV DNA in whole blood observed in the liver function impairment group (Table 3) further support the relationship between EBV and CD4+ T cells.

Our study demonstrated that co-infections significantly prolonged the hospital stay and fever duration in children with EBV-related IM, accounting for 13.9% of cases, which warrants clinical attention. Interestingly, patients with co-infection involving group A streptococcus (GAS) had a shorter hospital stay, which may be attributed to timely antimicrobial therapy and the current lack of GAS antibiotic resistance [26]. However, whether GAS acts as an infectious agent or merely a colonizer in these cases remains a subject of debate. This uncertainty is partly due to the fact that GAS can persist in the pharynx for extended periods without causing obvious infection or inflammation [27]. Furthermore, the interaction between viruses and bacteria likely plays a significant role in these infections, but the specific mechanisms underlying this interaction require further investigation [28]. In children, GAS infections typically manifest as pharyngitis and tonsillitis, but they can also progress to more severe conditions, such as invasive GAS infections, which include deep soft tissue infections, bacteremia, and streptococcal toxic shock syndrome [29]. In our study, no cases of invasive streptococcal infection were identified; all GAS-related cases presented as tonsillar abscesses.

Despite widespread off-label use of acyclovir in uncomplicated pediatric EBV-IM (49.33% in this study), our data corroborate systematic evidence demonstrating no reduction in fever duration or hospitalization with antiviral monotherapy. Supporting this, the research, including a systematic review [30], indicates that antiviral therapy has minimal impact on shortening illness or fever duration in EBV-IM patients and lacks significant overall clinical benefit, despite possible symptom relief in specific cases. Furthermore, the effectiveness of antiviral therapy in EBV infections, particularly among immunocompetent patients, remains uncertain, with studies showing that the suppression of viral replication is transient and does not persist post-treatment [31].This context of limited antiviral efficacy is further complicated by observations in the steroid group, where longer fever duration may reflect immune suppression delaying viral clearance [30], as the clearance of viral infections depends on a functional T-cell response [32,33]. Collectively, these findings challenge the routine use of antiviral therapy in uncomplicated EBV-IM and underscore the critical need for further investigation to definitively establish its role, if any, in clinical management.

Notably, studies show that EBV infection is associated with the development of various malignant tumors, particularly hematological malignancies and epithelial cell carcinomas [34]. In this study (Figure 1), during the acute phase, 15 children developed severe complications, including 13 with HLH and 2 with lymphoma. It should be noted that HLH is a hyperinflammatory syndrome rather than a malignancy, whereas lymphoma is a malignant condition. HLH patients exhibited severe leukopenia (median WBC 4.06 vs. 14.46 × 10^9^/L in IM; *p* < 0.05) without significant EBV DNA load differences (*p* < 0.05). The leukopenia observed in HLH patients can be attributed to the excessive immune activation and cytokine storms that are hallmarks of the disease, and we noted in our study that the percentage of CD4+ T cells decreased in the EBV-HLH patients when compared with healthy controls [24]. Despite this leukopenia, there was no significant difference in the EBV DNA load between HLH patients and those with IM, indicating that the viral load does not necessarily correlate with the severity of leukopenia in HLH [24]. Thus, studies show that changes in the immune system following EBV infection can influence disease progression over the long term [35]. Therefore, continuous monitoring of EBV load and immune status may help identify high-risk patients early and take appropriate preventive measures.

This study provides important insights into the clinical management of EBV-IM but has several limitations that should be carefully considered when interpreting the results. First, the retrospective design may introduce selection bias and limit the control of confounding factors. Additionally, the nature of observational studies means they can only reveal correlations between variables, not causality. While we identified significant associations between certain laboratory markers and clinical outcomes using statistical methods, these associations do not imply definitive causal relationships. Establishing causality would require more rigorous experimental designs. Second, the sample size, although relatively large, may still have limitations in detecting rare events or subgroup effects. Third, the assessment of treatment outcomes was limited to short-term effects. Long-term follow-up data are needed to evaluate the potential risks of antiviral therapy, such as the development of chronic active EBV infection. Lastly, the study was conducted in a single center, which may limit the generalizability of the findings to other populations.

## 5. Conclusions

In summary, our study of children with EBV-related IM at Shenzhen Children’s Hospital from 2009 to 2024 identified key markers like ferritin and LDH that predict severe outcomes. Co-infections, especially with GAS, influenced fever duration and hospital stay, suggesting personalized treatment is needed. Antiviral therapy showed limited benefit, indicating a need for re-evaluation of treatment strategies. Future research should focus on prospective studies to optimize management of EBV-IM.

## Figures and Tables

**Table 1:** Characteristics from May 2009 to April 2024 in Shenzhen Children’s hospital: inpatients with EBV-related IM (*n* = 2660).

Characteristic	Value	Range
Age, median [Q1, Q3] years	4 years [2, 6]	[0.67, 17]
<1 year	319 (12.0%)	
1–3 years	845 (31.8%)	
3–6 years	1007 (37.8%)	
6–17 years	489 (18.4%)	
Male: Female ratio	1578:1082 (1.46:1)	
Length of stay, *n* (%)		
Median [Q1, Q3]	5 (4, 7)	[1, 33]
<3 days	445	
3–7 days	1794	
8–14 days	401	
>14 days	20	
Febrile duration, *n* (%)		
Median [Q1, Q3]	7 (5, 9)	[1, 31]
<7 days	262 (52.4)	
7–14 days	210 (42.0)	
>14 days	28 (5.6)	

**Table 2:** Multivariate logistics regression analysis on the risk factors associated with fever duration >1 week and abnormal liver function to IM.

Variable	Fever Duration >1 Week			Variable	Abnormal Liver Function		
	OR	95% CI	*p*		OR	95% CI	*p*
Ferritin, ng/mL	1.004	1.002, 1.006	<0.001	CRP, mg/L	0.955	0.935, 0.976	<0.001
Atypical lymphocyte %	1.038	1.008, 1.070	0.012	LDH, u/L	1.004	1.002, 1.005	<0.001
				CD4%	0.894	0.856, 0.935	<0.001

**Table 3:** Comparison of WBC, EBV DNA load in whole blood and plasma among different patient groups.

Value	Comparison	Whole Blood EBV DNA^+^ (×10^3^ Copies/mL)		Plasma EBV DNA^+^ (×10^3^ Copies/mL)		WBC (×10^9^/L)	
		Median [Q1, Q3]	*p*	Median [Q1, Q3]	*p*	Median [Q1, Q3]	*p*
HLH or not	HLH	352.20 (16.02, 5493.50)	0.874	2.055 (0.6173, 37.8)	0.815	4.06 (2.44, 5.67)	0.015
	IM	242.00 (32.58, 1232.50)		3.39 (0.987, 12.35)		14.46 (9.82, 17.57)	
Fever Time	≥7 days	326 (27.30, 925.00)	1.000	1.92 (0.73, 4.42)	0.987	13.49 (11.10, 18.30)	0.99
	<7 days	314.00 (158.00, 1700.00)		2.01 (0.71, 4.70)		14.27 (10.50, 17.40)	
Liver function	Normal	187.00 (22.50, 901.00)	0.001	2.88 (0.846, 11)	0.085	13.40 (10.10, 17.30)	0.002
	Abnormal	340.50 (49.53, 1682.50)		3.73 (1.12, 13.85)		14.00 (10.80, 18.60)	

## Data Availability

The datasets used and/or analyzed during the current study are available from the corresponding author upon reasonable request.

## Supplementary Materials

The following supporting information can be downloaded at: https://www.mdpi.com/article/10.3390/pathogens14070702/s1, Table S1: Detailing all etiological diagnostic methods;Table S2:Clinical features from May 2009 to April 2024 in Shenzhen Children's hospital, inpatents with EBV related IM (n = 3006); Table S3: Comparison of Clinical Outcomes between Uncomplicated EBV-IM and Co-infections, including GAS (Subgroup Analysis); Table S4: Diagnostic Performance and Predictive Value of Risk factors; Table S5: Treatment Regimens in 1,729 Uncomplicated EBV-IM Cases [No Co-infections or Underlying Diseases]; Table S6: Comparison of EBV DNA Load and CBC in Whole Blood and Plasma Among Different Patient Groups.

## Abbreviations

AUC	Area under the curve
CAEBV	Chronic active EBV infection
CMV	Cytomegalovirus
CRP	C-reactive protein
EBV	Epstein–Barr virus
GAS	Group A streptococcus
MRSA	Methicillin-resistant staphylococcus aureus
IM	Infectious mononucleosis
IVIG	Intravenous immunoglobulin
LDH	Lactate dehydrogenase
ROC	Receiver operator characteristic
SPSS	Statistical Package for Social science.
RSV	Respiratory syncytial virus
WBC	White blood cell
