# Supplementary material for: Retrospective Review of Children Hospitalized for Epstein–Barr Virus-Related Infectious Mononucleosis
Source: Pathogens. 2025 Jul 16;14(7):702. doi: 10.3390/pathogens14070702 (PMC12299831; doi:10.3390/pathogens14070702)
Supplement: Supplementary file 1 [file pathogens-14-00702-s001.zip › pathogens-3729734-supplementary.pdf]

Supplementary Table S1. Detailing all etiological diagnostic methods.

Pathogen	Specimen Type	Detection Method	Platform/Technique
**Group A Streptococcus**	Throat swab	Rapid antigen test	Immunochromatographic assay
Helicobacter pylori	Exhaled breath	13 C-UBT	Infrared spectrophotometry
**Rotavirus**	Stool	Rapid Antigen Test	Colloidal gold method
**Cytomegalovirus [CMV]**	Blood	Quantitative PCR	Real-time PCR System [ABI 7500]
**Varicella-zoster virus**	Vesicle fluid	Real-time fluorescence PCR method	Real-time PCR System [ABI 7500]
**Amoebae**	Fresh stool	Microscopic examination	Saline/Iodine wet mount
**MRSA**	Sputum	Culture & identification	Mannitol salt agar + oxacillin
**Group G Streptococcus**	Sputum	Culture & identification	Blood agar + MALDI-TOF MS
**Other respiratory pathogens**	Nasopharyngeal swab	Multiplex PCR and capillary electrophoresis fragment analysis	Real-time PCR System [ABI 7500]

Abbreviations: 13 C-UBT, carbon-13 urea breath test; PCR , polymerase chain reaction; MRSA, methicillin-resistant Staphylococcus aureus; MALDI-TOF MS， matrix-assisted laser desorption ionization-time of flight mass spectrometry

Supplementary Table S2 Clinical features from May 2009 to April 2024 in Shenzhen Children's hospital, inpatents with EBV related IM（n=3006）

Clinical features	n[%]
Total	3006[%]
Fever	2499[93.9]
Cough	1442[54.2]
Abdominal pain	389[14.6]
Fatigue	76[2.9]
Nausea	54[2.0]
Vomiting	245[9.2]
Rhinorrhea	939[35.3]
Tonsillitis	2475[93]
Pharyngalgia	885[33.3]
Headache	106[4.0]
Arthralgia	10[0.4]
Myalgia	18[0.7]
Rash	294[11.1]
Jaundice	6[0.2]
Eyelids edema	1419[53.3]
Lymphadenopathy	2501[94]
Hepatomegaly	1938[72.9]
Splenomegaly	1457[54.8]

Supplementary Table S3. Comparison of Clinical Outcomes between Uncomplicated EBV-IM and Co-infections, including GAS(Subgroup Analysis)

Outcome Measure	Uncomplicated EBV-IM (n=1729)	Co-infection (n=368)	P-value	Co-infection with GAS (n=105)	*p*
**Liver Function Abnormality**	870(50.32%)	172 (46.7%)	0.212	59 (56.2%)	0.838
**Fever Duration (days)**	7(5,9)	7 (5,10)	<0.001	6(5,10)	<0.001
**Hospital Stay (days)**	5(4,6)	5 (4,7)	0.017	5(4,7)	0.217

Abbreviations:GAS, Group A Streptococcus

Supplementary Table S4 **Diagnostic Performance and Predictive Value of Risk factors**

S4-1 Risk factors for predicting the febrile course of infectious cytosis ≥7days

Risk factors	Cut-off Value	Sensitivity(%)	Specificity(%)	PPV	NPV	*P*	AUC	AUC(95% CI)
Atypical lymphocyte	22.45%	30.6	80.2	56.5	58.9	0.021	0.561	0.500-0.618
Ferritin	136.36ng/mL	44.5	82.9	85.2	73.6	0.001	0.662	0.588-0.729
Atypical lymphocyte+Ferritin	/	62.5	62.7	62.1	66.1	<0.001	0.679	0.607-0.746

S4-2 Prediction of risk factors for liver function impairment in infectious mononucleosis

Risk factors	Cut-off Value	Sensitivity(%)	Specificity(%)	PPV	NPV	*P*	AUC	AUC(95% CI)
CRP	10.69mg/L	46.4	73.6	63.7	78.3	<0.001	0.627	0.583-0.712
LDH	496.5U/L	65.2	67.6	48.2	81.4	<0.001	0.713	0.685-0.747
CD4%	20	45.9	81.6	56.9	80.7	<0.001	0.670	0.591-0.758
CRP+LDH+CD4%	/	58.7	85.2	67.9	79.6	<0.001	0.779	0.735-0.823

Abbreviations: AUC, area under the ROC curve; ROC, receiver operator characteristic;PPV, Positive predictive value; NPV, Negative predictive value.

Supplementary Table S5. Treatment Regimens in 1,729 Uncomplicated EBV-IM Cases [No Co-infections or Underlying Diseases]

Treatment Regimen	n	% of Total	Fever Duration median [Q1,Q3]	Hospitalization median [Q1,Q3]
No medication [symptomatic support]	792	45.81%	7[5,9]	4[3,6]
Acyclovir monotherapy	699	40.43%	7[5,9]	5.0[4.0,7.0]
Acyclovir + Corticosteroids	67	3.88%	7[6,10.5]	6[5,7]
Acyclovir + IVIG	66	3.82%	10[8,15]	8[6,10]
Acyclovir + IVIG + Corticosteroids	21	1.21%	8[6,12]	9[7,12]
IVIG monotherapy	47	2.72%	12[8,13]	6[5,7]
IVIG + Corticosteroids	8	0.46%	8[6,11]	7[6,7]
Corticosteroids monotherapy	29	1.68%	7[5.5,10.5]	5[4,6]
Total	1,729	100.00%	7[5,9]	5[4,7]

Abbreviations: IVIG, Intravenous immunoglobulin.

Supplementary Table S6. Comparison of EBV DNA Load and CBC in Whole Blood and Plasma Among Different Patient Groups

Value	Comparison	Whole Blood EBV DNA^{+} ($\times 10^3$copies/mL)		Plasma EBV DNA^{+} ($\times 10^3$copies/mL)		WBC ($\times 10^9$/L)		HB（g/L)		PLT($\times 10^9$/L)		CRP(mg/L)		ESR(mm/H）	
		Median [Q1,Q3]	*p*	Median [Q1,Q3]	*p*	Median [Q1,Q3]	*p*	Median [Q1,Q3]	*p*	Median [Q1,Q3]	*p*	Median [Q1,Q3]	*p*	Median [Q1,Q3]	*p*
HLH or not	HLH	352.20(16.02,5493.50)	0.874	2.055(0.6173,37.8)	0.815	4.06(2.44,5.67)	0.015	116.0(92.0,117.0)	0.013	102.0(60.0,212.0)	0.004	5.00(1.60,13.10)	0.696	20.9(6.4,28.3)	0.161
	IM	242.00(32.58,1232.50)		3.39(0.987,12.35)		14.46(9.82,17.57)		119.0(112.0,125.0)		217.0(173.0,264.0)		6.07(2.27,13.94)		24.9(17.7,33.8)	
Fever time	≥7days	326(27.30,925.00)	1.000	1.92(0.73,4.42)	0.987	13.49(11.10,18.30)	0.99	120.0(113.0,127.3)	0.002	208.0(169.0,245.5)	0.560	26.300(20.1,34.2)	<0.001	26.3(20.1,34.2)	<0.001
	＜7days	314.00(158.00,1700.00)		2.01(0.71,4.70)		14.27(10.50,17.40)		117.0(111.0,123.0)		213.0(168.0,264.0)		21.400(16.3,31.0)		21.4(16.3,31.0)	
Liver function	Normal	187.00(22.50,901.00)	0.001	2.88(0.846,11)	0.085	13.40(10.10,17.30)	0.002	119.0(112.00,125.00)	0.957	229.0(183.8,278.0)	<0.001	8.5(3.2,19.3)	<0.001	27.6(20.0,37.3)	<0.001
	Abnormal	340.50(49.53,1682.50)		3.73(1.12,13.85)		14.00(10.80,18.60)		119.0(112.00,126.00)		206.0(166.0,251.3)		4.7(1.6,10.5)		22.1(16.0,29.9)	

Abbreviation: CBC, complete blood count; WBC, white blood cell; HB, hemoglobin; CRP, C-Reactive Protein
